# Predisposing factors for poor outcome of surgery for cervical spondylotic amyotrophy: a multivariate analysis

**DOI:** 10.1038/srep39512

**Published:** 2016-12-19

**Authors:** JingTao Zhang, Can Cui, Zhao Liu, Tong Tong, RuiJie Niu, Yong Shen

**Affiliations:** 1 Department of Spinal Surgery, The Third Hospital of Hebei Medical University, Shijiazhuang, Hebei, People’s Republic of China; 2 Department of Neurology, The Second Hospital of Hebei Medical University, Shijiazhuang, Hebei, People’s Republic of China

## Abstract

The purpose of this study was to characterize risk factors for poor surgical outcome in patients with cervical spondylotic amyotrophy (CSA). We retrospectively reviewed 88 cases of CSA surgery and investigated age, sex, duration of symptoms, atrophy type, preoperative muscle power, signal changes on MRI, anterior horn (AH) or ventral nerve root (VNR) compression, compression levels, surgical approach and postoperative recovery. Fifty (56.8%) patients had good surgical outcome. Logistic regression, with poor outcome as dependent variable, showed independent risks associated with duration of symptoms (OR; 1 for symptom duration less than 3 months versus 3.961 [95% CI; 1.203–13.039, p = 0.024] for symptom duration of 3–6 months versus 18.724 [95% CI; 3.967–88.367, p < 0.001] for symptom duration greater than 6 months), compression type (OR; 1 for VNR versus 4.931 [95% CI; 1.457–16.685, p = 0.010] for AH versus 5.538 [95% CI; 1.170–26.218, p = 0.031] for VNR + AH), and atrophy type (OR; 1 for proximal type versus 6.456 [95% CI; 1.938–21.508, p = 0.002] for distal type). These findings suggest that a long duration of symptoms, AH or both AH and VNR compression, and distal type are risk factors for poor surgical outcome in patients with CSA.

Cervical spondylosis often manifests with spastic tetraparesis with varying degrees of sensory dysfunction^1^,^2^; however, in rare cases, it causes muscle atrophy of the upper extremities without sensory disturbance or pyramidal signs. This phenomenon was first reported by Keegan^3^ as “dissociated motor loss in the upper extremities with cervical spondylosis”. Cervical spondylotic amyotrophy (CSA) was first reported by Sobue *et al*.^4^ in 1975 as a subtype of cervical spondylotic myelopathy. It is characterized by muscle weakness and atrophy in the upper extremities with no or insignificant sensory deficit and gait disturbance^5^. The upper extremity muscle atrophy caused by CSA is generally classified into two subgroups according to the most predominantly affected muscles: proximal type (scapular, deltoid and biceps muscles) and distal type (triceps, forearm and hand intrinsic muscles)^4^,^6^. Difficulty in shoulder abduction, positive arm-drop sign, or positive wrist-drop sign is always found in patients with CSA^7^. It is usually reported as unilateral disorder, and occasionally, it presents as bilateral^8^. Two different mechanisms have been proposed for the pathophysiology of CSA. Selective damage to either ventral nerve root (VNR) or anterior horn (AH) may cause CSA^3^,^5^,^7^. Alternatively, the mechanism of vascular insufficiency to the AH cells at the paramedian compression has also been proposed^4^.

Usually, patients with CSA are first treated conservatively, such as with cervical traction or wearing a neck collar. Failed to response to conservative treatment and a definite diagnosis of CSA were considered indications for surgery^9^,^10^,^11^. However, even with surgical treatment, some patients may not benefit from surgery^7^,^9^,^11^,^12^,^13^,^14^. There are a few studies investigate risk factors of poor surgical outcome for CSA^10^,^11^,^15^,^16^, but many of these studies have been limited by a small sample size, which has restricted their investigation to a few potential risk factors, and most lack the power to perform a multivariate analysis. As we know, the essential pathophysiologies of the two compression types are obviously different. The purpose of this study was to conduct a large retrospective study and multivariate analysis to assess previously indentified risk factors as well as to report novel risk factors of poor surgical outcome for CSA patients.

## Materials and Methods

### Ethics statement

The study was approved by Ethics Committee of the Third Hospital of Hebei Medical University in China. There is no need to obtain informed consent from patients since this is a retrospective study and all the data were collected and analyzed anonymously. The methods were carried out in accordance with the approved guidelines.

### Study design and patient population

This retrospective study included data of patients who underwent surgery for CSA at the Department of Spinal Surgery, the Third Hospital of Hebei Medical University in China, between January 2000 and December 2014. Diagnosis was confirmed by clinical manifestations, radiological findings, computed tomography myelography (CTM) or magnetic resonance imaging (MRI), electromyogram (EMG), nerve conduction testing, and segmental spinal cord evoked potentials. The inclusion criteria were as follows: presence of severe atrophy of the upper extremity muscles; unilateral upper extremity involved; insignificant or no sensory deficit in the upper extremity; absence of gait disturbance, spasticity of lower limbs or deep tendon hyperreflexia; absence of pyramidal signs such as Bakinski sign or Oppenheim sign; presence of MRI-documented spinal cord compression and/or nerve root compression. Exclusion criteria were vitamin B deficiency, motor neuron diseases (MND), muscular dystrophy, collagen disease, or torn rotator cuff lesions, cervical ossification of posterior longitudinal ligament and previous cervical spine surgery.

All enrolled patients underwent surgical decompression to regain the muscle strength of upper extremity. The surgical approach and the number of operated segments were determined by one surgeon (Y.S.). Patients treated anteriorly underwent cervical discectomy and fusion, or cervical corpectomy and fusion. Posterior procedures included laminoplasty with or without foraminotomy. Operative selections for CSA patients were based on the number of intervertebral levels involved: (1) Up to three levels: anterior surgery; (2) three or more levels: posterior surgery.

### Assessment of surgical outcome

Muscle strength was evaluated by manual muscle testing (MMT) using the 6-point Medical Research Council scale. Compared with contralateral upper extremity, we confirmed a single muscle as the most severely atrophic and impaired muscle. To evaluate the effect of surgical treatment at 1 year of follow-up, we used MMT, and improvements in the muscle strength of the most severely atrophic and impaired muscle were classified in 4 grades: excellent, more than 2 scores of recovery on MMT; good, 1 score of improvement on MMT; fair, no improvement on MMT; poor, worsening on MMT. Grades of excellent and good neurologic recovery were considered as “good outcome”, and grades of fair and poor as “poor outcome”.

### Relevant factors

**Figure 1:**
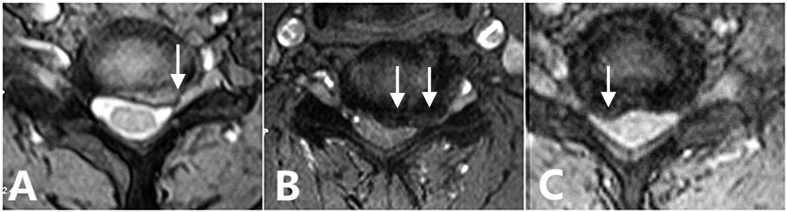
(**A**) Axial T2-weighted MRI showed ventral nerve root (VNR) compression at the C6–7 intervertebral foramen. (**B**) Axial T2-weighted MRI showed anterior horn (AH) compression at the paramedial site of spinal canal behind the C6–7 intervertebral level. (**C**) Axial T2-weighted MRI showed both AH and VNR were compressed at the C5–6 intervertebral level.

Investigators collected data including age, sex, duration of symptoms, atrophy type, preoperative MMT score, surgical approach and imaging-related factors such as the number of intervertebral levels involved, low signal on T1 MRI, high signal on T2 MRI and compression type. Patients with AH compression were characterized by spinal cord lesions at the medial or paramedial site of spinal canal with or without signal intensity changes on sagittal MRI. However, patients with VNR compression had nerve root lesions alone at the intervertebral foramen on T2-weighted axial view, with no signal intensity changes on T1- or T2-weighted sagittal view. Both AH and VNR compressions were also showed in our patients (Fig. 1).

### Statistical analysis

Descriptive analysis of the patient population was conducted using means and standard deviations (SD) for continuous variables and frequencies and percentages for categorical and discrete variables. All potential risk factors were evaluated for a univariate association with poor surgical outcome, with use of independent-samples t tests for continuous variables and chi-square or Fisher exact tests for categorical or discrete variables. A multivariate logistic regression was then used to evaluate the independent associations of each potential explanatory variable. Factors with a p-value of less than 0.05 in univariate analysis were entered into the multivariate logistic model. Adjusted odds ratios (OR) with 95% confidence intervals (CI) were presented with their respective p-values. A value of P < 0.05 was considered to represent a statistically significant difference. All analyses were performed using SPSS software (version 21.0; SPSS Inc., Chicago, IL, USA).

## Results

### General data of patients included

A total of 88 patients with CSA were ultimately included in this study. There were 52 men and 36 women, ranging in age from 42 to 82 years, with a mean age of 61 years. The mean duration of symptoms was 10.8 months, ranging from 0.5 months to 9 years. The mean follow-up period was 5.2 years, ranging from 1 to 13 years. Fifty-two patients had proximal-type CSA, and 36 patients had distal-type CSA. The compression type were VNR compression for 45 patients, AH compression for 27 patients and both for 16 patients. High signal on T2 MRI was confirmed in 21 patients, and low signal on T1 MRI was confirmed in five patients. Compression segments were found at an average of 1.8 intervertebral levels, ranging from 1 to 4 levels. Sixty patients received anterior cervical discectomy and fusion; nine patients received anterior cervical corpectomy and fusion; six patients received posterior laminoplasty; thirteen patients received laminoplasty with foraminotomy. Generally, the muscle strength of the most atrophic and impaired muscle was significantly improved. The mean MMT score increased to 3.1 at the last follow-up, significantly higher than 2.2 before surgery (p < 0.01). The surgical outcomes were excellent for 31 patients, good for 19, fair for 37 and poor for 1. Fifty patients had good surgical outcome, with MMT score improved greater than or equal to 1, while 38 patients had poor surgical outcome with no MMT score improvement or worsening.

### Comparison of clinical and radiological data

According to the univariate analysis, advanced age was associated with poor postoperative outcome, and within the groups the mean age was 63.7 years for the poor outcome patients and 59.5 years for the good group (p = 0.024). Low preoperative MMT score was also associated with poor outcome, and within the groups the mean preoperative MMT score was 1.9 for the poor outcome patients and 2.4 for the good group (p = 0.021). Also in the univariate analysis, distal type was a significant risk factor for poor surgical outcome, as 55% of the poor group was distal type (p = 0.017). Similarly, duration of symptoms and compression type were risk factors for poor postoperative outcome (p = 0.001, p = 0.001, respectively). There were no significant differences between the two groups regarding sex, the number of intervertebral levels involved, low signal on T1 MRI, high signal on T2 MRI, and surgical approach (Table 1).

### Multivariate logistic regression analysis

In multivariate logistic regression analysis, duration of symptoms (OR; 1 for symptom duration less than 3 months versus 3.961 [95% CI; 1.203–13.039, p = 0.024] for symptom duration of 3–6 months versus 18.724 [95% CI; 3.967–88.367, p < 0.001] for symptom duration greater than 6 months), compression type (OR; 1 for VNR versus 4.931 [95% CI; 1.457–16.685, p = 0.010] for AH versus 5.538 [95% CI; 1.170–26.218, p = 0.031] for VNR + AH), and atrophy type (OR; 1 for proximal type versus 6.456 [95% CI; 1.938–21.508, p = 0.002] for distal type) were identified as independent predictors of poor postoperative outcome. Age at operation and preoperative MMT score were significant in the univariate analysis, but did not meet significance in the final logistic regression model (Table 2).

## Discussion

The elucidation of factors that contribute to prognosis of CSA patients has been investigated by several groups^10^,^11^,^15^,^16^. Recognition of the best timing for surgery to ensure neurological recovery is an important clinical issue. The focus of our study was to further define significant risk factors that contributed to a higher rate of poor outcome after the surgical treatment of CSA. The results of this study showed that a longer duration of symptoms, AH involvement and distal type were significant independent risk factors for poor surgical outcome.

Duration of symptoms has consistently been reported as a risk factor for poor outcome following surgery^15^,^16^. The duration of symptoms affects the severity and progression of the disease due to chronic compression by the lesions. The rationale is that long-standing and chronic compression of the spinal cord or nerve root may lead to irreversible damage due to demyelination and necrosis of the gray matter. Therefore, to achieve the best results, surgical intervention should be undertaken as early as possible. Similarly, Uchida *et al*.^10^ reported that there was a close association between duration of disease and improvement of muscle power in both subgroups. Therefore, they suggested that surgical treatment of CSA requires urgent action.

Additionally, AH compression or both the AH and VNR compression were independent risk factors for poor surgical outcome. This association has not been noted in other studies to date, as far as we know. Therefore, compression type on MRI is a novel prognostic factor when making decisions on treatment strategy. The spinal cord, including the AH, has less ability to regenerate compared with the VNR. Maybe it could explain why a patient with AH compression or both AH and VNR compression is at increased risk for poor recovery.

Most significantly our study results suggested that the response to surgery was different in the two atrophy types of CSA. The surgical outcome for distal-type CSA was inferior to the outcome for proximal-type CSA (OR, 6.456; p = 0.002). Fujiwara *et al*.^9^ reported that muscle power on MMT improved in 92% of the proximal-type CSA but only improved in 38% of the distal-type CSA. Zhang *et al*.^17^ reported that 81% of proximal-type patients gained 1 or more scores of muscle strength improvement on MMT, whereas only 40% of distal-type patients improved. Interestingly, Kaneko *et al*.^18^ reported that the pathophysiology of distal-type CSA included widespread AH lesions with less involvement of lateral VNR. Uchida *et al*.^10^ also reported that the distal type showed a greater number of involved vertebrae causing cord compression and a poorer outcome than the proximal type. By literatures review, we can find two possible reasons to interpret the distinct surgical outcomes^9^,^19^. One possible reason is the longer distance from the cervical spinal cord or nerve root to the atrophic muscles in distal type than in proximal type. The other possible reason is that distal type basically involves impingement against the AH which has less ability than the VNR to regenerate.

Previous report also suggested that preoperative MMT score was associated with a poor postoperative outcome in CSA patients^15^. This could be attributable to the fact that neurological recovery of the spinal cord or nerve root depends on irreversible change. Although our univariate analysis indicated that preoperative MMT score was associated with a poor surgical outcome, it did not meet significance in the final logistic regression model. Similarly, Uchida *et al*.^10^ reported that the improvement of muscle strength did not correlate with preoperative MMT score. However, Musha and Mizutani^20^ reported that patients with mild paralysis tended to improve better than those with severe paralysis when they received conservative treatment. This discrepancy could lie in the disability of preoperative MMT score to reflect the regenerative potential of spinal cord or nerve root. For example, a young CSA patient with muscle atrophy of acute onset had a satisfactory surgical effect, even if the preoperative MMT score was low.

In the present study, signal intensity changes on T2 or T1 MRI and the number of intervertebral levels involved were not significantly associated with a poor postoperative outcome. High signal change on T2-Weighted MRI reflected edema, myelomalacia, and that low signal change on T1-Weighted MRI indicated cystic necrosis or secondary syrinx^21^. The pathology of this signal abnormality has been assumed to vary from acute edema to chronic myelomalacia, and is associated with a poor postoperative outcome of cervical spondylotic myelopathy^22^,^23^,^24^,^25^. Despite such evidence, our study found no correlation between and a poor surgical outcome and intramedullary signal intensity change on MRI. A possible reason for this phenomenon is that the intramedullary signal intensity change is usually consistent with lesion of the central portion of spinal cord, which can not reflect the pathological changes of AH.

The appropriate surgical methods for CSA are controversial^7^,^9^,^12^,^14^,^17^,^26^,^27^,^28^,^29^,^30^. Surgical approaches such as anterior cervical decompression and fusion or laminoplasty with or without foraminotomy have been advocated. To date, there appears no significant difference in postoperative outcome between the two surgical procedures. Surgical approach was not significant independent risk factors identified by our multivariate analysis, a finding consistent with other analyses. The anterior surgery is believed to provide an optimal neurological recovery through the complete elimination of the compression, but can be rather technically demanding when the compression is at the foraminal entrance and at three or more intervertebral levels. Posterior decompression with foraminotomy has the disadvantage of leaving the anterior compressive lesion as is, although this procedure is less technically demanding. In our study, compressions involving 1 intervertebral level are most, followed by 2-level and 3-level compressions. Therefore, anterior surgery was the most popular and effective operation for our study patients.

Several limitations of our study must be acknowledged. First, a retrospective study is the most practical way to study the relatively rare occurrence of disease; however, its use implies certain inherent limitations, such as possibility of underestimating the incidences of poor surgical outcome. Second, previous studies demonstrated that motor-evoked potentials and somatosensory evoked potentials are useful in monitoring the effectiveness of surgery for patients with cervical spondylotic myelopathy^31^,^32^; however, the study did not include an analysis of the electrophysiological data due to the lack of consistency. Third, the study analysis did not include comorbidities such as diabetes or smoking which could play any role in surgical outcome.

The strengths of our study include the large volume of CSA surgeries performed at a single institution, allowing for a large sample and thereby increasing the power of analysis. This study is one of the first with enough power to conduct a multivariate analysis of risk factors to better assess their contribution to poor postoperative outcome for CSA. The analysis confirms previously demonstrated risk factors for poor outcome while identifying new potential independent risk factors such as compression type. Areas for continued study include the role of comorbidities and electrophysiological examination in CSA patient surgery.

## Additional Information

**How to cite this article**: Zhang, J.T. *et al*. Predisposing factors for poor outcome of surgery for cervical spondylotic amyotrophy: a multivariate analysis. *Sci. Rep.* **6**, 39512; doi: 10.1038/srep39512 (2016).

**Publisher's note:** Springer Nature remains neutral with regard to jurisdictional claims in published maps and institutional affiliations.

## Figures and Tables

**Table 1:** Comparison of patient characteristics between good and poor outcome groups.

Variable	Good (n = 50)	Poor (n = 38)	P-value
Age at operation (yr)	59.5 ± 8.5	63.7 ± 8.4	0.024
Male sex (n, %)	33 (66.0%)	19 (50.0%)	0.131
Duration of symptoms (mo)			
＜3	33	10	0.001
3–6	12	15	
>6	5	13	
Preoperative MMT score	2.4 ± 1.0	1.9 ± 1.1	0.021
Atrophy type			
Proximal type	35	17	0.017
Distal type	15	21	
Levels involved	1.7 ± 0.7	2.0 ± 0.9	0.086
Compression type			
VNR	31	14	0.001
AH	10	17	
VNR + AH	9	7	
Low signal on T1 MRI	3 (6.0%)	2 (5.3%)	1.000
High signal on T2 MRI	10 (20.0%)	11 (29.0%)	0.366
Surgical approach			
Anterior	39	30	0.915
Posterior	11	8	

MMT: manual muscle testing; AH: anterior horn; VNR: ventral nerve root; MRI: Magnetic resonance imaging; mo: month; yr: year.

**Table 2:** Risk factors for poor surgical outcome: multivariate logistic regression analysis.

Variable	OR (95% CI)	P-value
Age at operation (yr)	1.012 (0.949–1.079)	0.719
Preoperative MMT score	0.613 (0.321–1.171)	0.139
Atrophy type		
Proximal type	1	
Distal type	6.456 (1.938–21.508)	0.002
Duration of symptoms (mo)		
<3	1	
3–6	3.961 (1.203–13.039)	0.024
>6	18.724 (3.967–88.367)	<0.001
Compression type		
VNR	1	
AH	4.931 (1.457–16.685)	0.010
VNR + AH	5.538 (1.170–26.218)	0.031

MMT: manual muscle testing; AH: anterior horn; VNR: ventral nerve root; OR: odds ratio; CI: confidence interval; mo: month; yr: year.
